# Supplementary material for: Opening and closing of a cryptic pocket in VP35 toggles it between two different RNA-binding modes
Source: eLife. 2025 Sep 2;14:RP104514. doi: 10.7554/eLife.104514 (PMC12404614; doi:10.7554/eLife.104514)
Supplement: Supplementary file 1. [file elife-104514-supp1.docx]

|  |  | C296 |  |
| --- | --- | --- | --- |
| Homolog | k_open_  (s-1) | k_close_  (s-1) | k_int_  (μM ^-1^ s^-1^) |
| Reston WT | 0.7652002 $\pm$0.0000004 | 9.7$\pm$0.1 | 0.177 $\pm$0.003 |
| Reston P280A | 2.45 $\pm$ 0.07 | 1.19 $\pm$ 0.01 | 0.047 $\pm$ 0.005 |
| Zaire WT | 3.17 $\pm$ 0.07 | 7.66 $\pm$ 0.03 | 0.0136 $\pm$ 0.0003 |
| Zaire A291P | 0.62 $\pm$ 0.04 | 54.63 $\pm$ 0.05 | 0.081 $\pm$ 0.007 |
| Marburg WT | 2.5 $\pm$ 0.2 | 0.48 $\pm$ 0.005 | 0.0027 $\pm$ 0.0002 |

Supplementary Table 1: Estimates for opening (k_open_), closing (k_close_) and intrinsic labeling (k_int_) rates obtained from fits of observed labeling rates as a function DTNB concentration to the Linderstrøm-Lang model.
